# Mechanical and structural properties of major ampullate silk from spiders fed carbon nanomaterials

**DOI:** 10.1371/journal.pone.0241829

**Published:** 2020-11-09

**Authors:** Sean P. Kelly, Kun-Ping Huang, Chen-Pan Liao, Riza Ariyani Nur Khasanah, Forest Shih-Sen Chien, Jwu-Sheng Hu, Chung-Lin Wu, I-Min Tso

**Affiliations:** 1 Department of Life Science, Tunghai University, Taichung, Taiwan; 2 Mechanical and Mechatronics Systems Research Laboratories, Industrial Technology Research Institute, Hsinchu, Taiwan; 3 Museum of Natural Science, Taichung, Taiwan; 4 Department of Applied Physics, Tunghai University, Taichung, Taiwan; 5 Center for Measurement Standards, Industrial Technology Research Institute, Hsinchu, Taiwan; Universiti Teknologi Petronas, MALAYSIA

## Abstract

The dragline silk of spiders is of particular interest to science due to its unique properties that make it an exceptional biomaterial that has both high tensile strength and elasticity. To improve these natural fibers, researchers have begun to try infusing metals and carbon nanomaterials to improve mechanical properties of spider silk. The objective of this study was to incorporate carbon nanomaterials into the silk of an orb-weaving spider, *Nephila pilipes*, by feeding them solutions containing graphene and carbon nanotubes. Spiders were collected from the field and in the lab were fed solutions by pipette containing either graphene sheets or nanotubes. Major ampullate silk was collected and a tensile tester was used to determine mechanical properties for pre- and post-treatment samples. Raman spectroscopy was then used to test for the presence of nanomaterials in silk samples. There was no apparent incorporation of carbon nanomaterials in the silk fibers that could be detected with Raman spectroscopy and there were no significant improvements in mechanical properties. This study represents an example for the importance of attempting to replicate previously published research. Researchers should be encouraged to continue to do these types of investigations in order to build a strong consensus and solid foundation for how to go forward with these new methods for creating novel biomaterials.

## Introduction

Carbon nanomaterials have been a major focus of scientific research beginning with the first discovery of fullerene allotropes in the late 1980s [1] and in particular since the isolation and extraction of 2D graphene sheets in 2004 [2]. Due to their unique thermal, structural and mechanical properties, there have been numerous applications of graphene sheets (GS) and its allotropes such as carbon nanotubes (CNTs) in the fields of electronics and medicine [1, 3–5]. GS formed into CNTs have been shown to be the strongest synthesized material, with a strength two orders of magnitude greater than steel at only 1/6^th^ the weight [1, 3, 4]. The ability to combine the strongest known synthesized material (e.g., graphene and nanotubes) with the toughest known biological materials in nature (e.g., silkworm silk, spider silk) could lead to the creation of remarkable new bio-synthetic super materials [3, 6–8]. However, most of this research is still at the preliminary stages of proof of concept and results vary between experiments.

Silk from Mulberry Silkworm (i.e., *Bombyx mori*) cocoons and spider silk have been a focus for material science because of their incredible tensile properties and biocompatibility [3, 6, 7, 9–12]. The unique mechanical properties are a result of the combination of protein structures that include semi-amorphous α-helix regions that contribute to the silk’s elasticity and crystalline β-sheets that give the material its strength [9, 13–17]. The exact way the protein structures auto-align themselves into these super biomaterials is still poorly understood and is a topic of ongoing research [12, 15, 17, 18]. *B*. *mori* have been domesticated for thousands of years and the ability to collect silk at industrial scales makes this a popular biomaterial for creating nanocomposites [9, 17]. For an example, after feeding *B*. *mori* larva mulberry leaves coated with solutions containing carbon nanotubes (CNTs), silk from their cocoons was found to have double the toughness [6–8] and showed an increase in conductivity by more than 70% [7]. However, the drag line silk of orb-weaving spiders, known as major ampullate (MA) silk, has an even higher tensile strength of around 1000–1400 Mpa and an elasticity of around 20–30% [9–12, 16, 17]. This combination makes it one of the toughest materials known to science and around 200% tougher than that of *B*. *mori* silk [9, 17]. Therefore, recent work has focused on how to replicate successes observed in *B*. *mori* silk research with spider silk [3, 19].

Two methods used to combine spider silk fibers with carbon nanomaterials have been by either coating the exterior of the silk [11, 12] or incorporating the carbon nanomaterials into the silk by having spiders ingest a solution containing GS or CNTs [7]. For an example, in a study by Steven et al. (2013), major ampullate silk from *Nephila clavipes* spiders was coated with amine-functionalized multi-walled carbon nanotubes (f-NTS-SS) through the process of hydrating the silk and mechanically shearing the two materials together. Using this process, they were able to produce a tough, and flexible fiber that was also electrically conductive [20]. This study found that the ultimate strength of the f-NTS-SS fibers was less than that of natural silk fibers, however there was an increase in extensibility and therefore overall toughness was similar [20]. A study by Lepore et al., [19] presented data in which they believed they were able to show the presence of carbon nanomaterials in the silk of web-spinning spiders that resulted in the improvement of the silks’ tensile properties. However, in Lepore et al., [19] there was a high degree of variation in the values of tensile properties and there are some concerns over the methods used in the study. Values of tensile properties rely heavily on the ability to accurately measure the diameter of individual silk threads. In the study by Lepore et al., [19] silk diameter was accomplished by dividing the overall diameter of silk bundles and then divided this by the number of threads in each bundle, which ranged from a minimum of 4 to a maximum of 82. By measuring tensile properties of whole silk bundles, but calculating final values based on average silk thread diameters could lead to misinterpretations of the overall changes in tensile properties attributed to carbon nanomaterials. The detection of carbon nanomaterials through Raman spectra analyses in the study by Lepore et al., [19] could be a result of silk bundles being collected from the same containers where solutions containing GS and CNTs were sprayed. We feel it is necessary to attempt to replicate the proof of concept that is presented by Lepore et al., [19], but with improved methods that will ensure more accurate measurements of tensile properties and to remove any possibility for the contamination of silk fibers with CNT and GS solutions. It is important to validate the findings reported within the Lepore et al., [19] study before future work will be able to make any significant advances in the creation of new biomaterials involving carbon nanomaterials incorporated within the spider silk matrix.

The objective of our study was to incorporate carbon nanomaterials (i.e. graphene sheets and single-walled nanotubes) into the matrix of major ampullate silk fibers of *Nephila pilipes* (Giant Wood Spider). It was hypothesized that by feeding spiders solutions with similar concentrations of carbon nanomaterials to what were used by Lepore et al., [19], it would be possible to replicate the results of their study by showing increases in tensile properties of silk and confirming the presence of GS and SWNT with Raman spectra analyses. By replicating the results of Lepore et al., [19] with a species of spider that creates one of the toughest silks in nature, our aim was to produce a novel biomaterial with improved tensile properties that could be of great interest to a range of scientific fields and lead to practical applications for creating new biomedical and textile products.

## Materials and methods

The focal species chosen for this study was *Nephila pilipes* (Fabricius, 1793). *N*. *pilipes* is common throughout Southeast Asia and is one of the largest orb-weaving spiders in the world. The silk of *Nephila* spiders has been extensively studied, with the majority of research involving its mechanical properties [21–23] and biomolecular structures [15, 24]. Adult female *N*. *pilipes* were collected from local areas around central Taiwan and then housed in 20 x 30 cm wooden cages at Tunghai University. Spider size is associated with silk diameter, which is an important component when calculating mechanical properties. Therefore, data was collected on morphological characteristics of all spiders used in the study to test for any differences between treatment groups. Upon arriving to the lab all spiders were weighed and photographed for body size measurements (i.e. carapace width and total body length). Photographs of each spider were taken on 1 cm^2^ grid paper and measurements were done with the computer software ImageJ [25]. To minimize any effects from the field all individuals were allowed to acclimate in cages for five days and were daily given 50 μl of a protein solution which comprised of sucrose (0.2 g ml^-1^) dissolved in deionized water and then mixed with egg albumin (0.16 g ml^-1^). This protein solution was adopted from previous feeding experiments with orb-weaving spiders and was shown to be a suitable replacement for live prey [26]. On the sixth day, single strands of MA silk were collected from each spider for pre-treatment mechanical tests (e.g., Young’s modulus, toughness, ultimate strength and extensibility) and Raman Spectroscopy.

Three rounds of experiments were conducted with the objective of incorporating carbon nanomaterials into the major ampullate silk of *N*. *pilipes* spiders. 1) Two types of graphene sheets (GS) were used in the first experiment, 2) two types of single-walled nanotubes (SWNT) were used in the second experiment and 3) in the third experiment the concentration was doubled (2x) for one type of GS and one type of SWNT. A control group was included in each of the three experiments to take into account any unintended effects during the study from the feeding and housing of the spiders.

1) Graphene sheets (GS): 36 spiders were collected and two experimental groups of spiders (N = 12 per group) received 50 μl of graphene sheet (GS) solutions daily with each group receiving one of two different types of graphene sheets (MPT and RPS) which differed from each other by their number of graphene layers. MPT graphene was produced using a Microwave Plasma Torch method and contained ~3–5 layers, while RPS was produced using a Transformer Coupled Plasma method and contained ~10 layers.

2) Single-walled nanotubes (SWNT): 18 spiders were collected and assigned randomly to a control group and two treatment groups (N = 6 per group). The two treatment groups received 50 μl of solutions daily containing two types of single walled nanotubes (SWNT) (CT and T) which differed from each other by the length and level of imperfection of the nanotube structure. Differences in SWNT structures are a result of slight changes in the method in the catalytic decomposition of hydrocarbons [27].

3) Double concentration (2x): 42 spiders were collected and assigned randomly to a control group and two treatment groups (N = 14 per group). The two treatment groups received 100 μl of solutions daily containing one type of GS (MPT) and one type of SWNT (CT).

Treatment solutions containing the different carbon nanomaterials were adapted from Lepore et al., [19]. GS and SWNT solutions comprised of 20 mg ml^-1^ of sodium deoxycholate (SDC) dissolved in deionized water with the addition of 10 mg ml^-1^ of either GS (MPT and RPS) or SWNT (CT and T). Solutions were then placed into an ultrasonicator for ~12 hours in order to ensure a homogeneously mixed solution. Control groups received 50 μl of a solution containing 20 mg ml^-1^ of sodium deoxycholate (SDC) dissolved in deionized water but without carbon nanomaterials. In GS and SWNT experiments, spiders received 50 μl of the associated GS (MPT and RPS), SWNT (CT and T) or control solution for five days. In the 2x experiment, spiders received 100 μl of MPT, CT or control solution for three days. Post-treatment silk samples for mechanical tests and Raman Spectroscopy analyses were collected on the sixth day for the GS and SWNT experiments and on the fourth day for the 2x experiment.

### Mechanical tests

Spiders were transferred from their cages and affixed to foam platforms using non-adhesive tape and pins to make them immobile but without harming the spider. Using a dissecting microscope a single strand of MA silk was manually pulled from the anterior spinnerets, attached to a mechanical spool with masking tape and then reeled at a speed of 1 m min^-1^. A single strand of MA silk was collected on cardboard cards with three 10 mm gaps using a small amount of cyanoacrylate glue before and after each gap. Two cards were collected for each spider allowing for a total of six silk samples to be used for mechanical tests. Each card was taped to a glass slide and photographed at 100x using a digital camera (Canon EOS 650D) attached to a polarized light microscope. Photographs were used to measure the diameter of each silk thread with the program ImageJ [25], which were subsequently utilized in determining the mechanical properties of each silk fiber. Mechanical tests were conducted for each 10 mm silk sample using a Micro Bionix mechanical tester at the Center for Measurement Standards, Industrial Technology Research Institute, Hsinchu, Taiwan. Silks were stretched at a rate of 1% of the gauge length per second until rupture. Mechanical properties including: Young’s modulus, ultimate strength (i.e., true stress), extensibility (i.e., true strain) and toughness were derived from stress-strain curves plotted by the program Test Works 4.0.

### Raman spectroscopy

Raman spectroscopy is a technique to study vibrational, rotational, and other states in molecular systems. Raman spectroscopy can provide a structural fingerprint by which molecules in the material can be identified. Raman spectroscopy has been a preferred method in characterizing molecular structures in silk research, including the study done by Lepore et al. [19]. The objective of our research was to follow similar protocols as that of Lepore et al. [19] and therefore similar detection methods were used in order to compare our results more accurately. Single strand silk samples for Raman spectroscopy analyses were collected at the same time as silk samples used for testing mechanical properties. Post-treatment silk samples from control and treatment groups from the three rounds of experiments: (1) GS, (2) SWNT and (3) 2x were collected on cardboard cards with 10 mm x 30 mm gaps. In addition to silk samples, there were also Raman spectra analyses of GS (MPT and RPS) and SWNT (CT and T) pure powder samples. All Raman spectroscopy analyses were conducted by the Mechanical and Mechatronics Systems Research Laboratories at the Industrial Technology Research Institute (ITRI) in Hsinchu, Taiwan. Samples were placed onto glass slides and analyzed with a DXR2 Raman Microscope (ThermoFisher Scientific) with a green laser excitation light source of 532 nm under 100x objective. Samples were analyzed within the Raman shift range of 200–3400 cm^-1^ and Raman spectra were recorded as functions of intensity and Raman shift.

### Statistical analysis

To test for possible differences in spider size (i.e., total length, carapace width, mass) between treatment groups a multivariate analysis of variance (MANOVA) was conducted in the statistical program PAST [28] for each of the three experiments. The three body size variables (total length, carapace width, mass) were tested for normality in each of the three experiments and were log transformed when necessary to meet the assumptions for parametric analyses.

For each experiment, tensile properties among treatments and samples were fitted with a multivariate general linear mixed-effect model by using the R statistical environment [29] and the package ‘brms’ version 2.7.0 [30]. In the analyses for each of the three experiments, treatment group (i.e., Control, GS or SWNT types), pre-/post-treatment samples and their interactions were included as fixed factors. Spider identity and pre-/post-treatment samples within spider identity were included as correlated random intercepts to achieve comparisons, while taking into account the spider identity and to cope with pseudoreplications. Weakly informative priors were assigned for all parameters (i.e., intercept term, 2 times scaled T distribution with DF = 3; fixed effect, 2 times scaled T distribution with DF = 7; random effect and residual, exponential distribution with rate = 1). 5,000 MCMC iterations (including the beginning 4,000 burn-in iterations) per thread were performed, and in total 10 threads were parallel proceeded for each parameter. Pair-wise Bayesian MCMC equivalence tests were conducted, and the region of practical equivalence (ROPE) was assigned as ±0.1 Cohen’s *d*. Only the posterior distribution of differences completely laying outside the ROPE were considered as significant. The full results of pair-wise Bayesian MCMC equivalence tests are included as S1 to S6 Tables.

## Results

### Spider body measurements

No significant difference in spider body size (i.e., carapace width, body length and mass) was detected between control and experimental groups in the GS (MANOVA, Wilks’ lambda: 0.85, F: 0.87, p = 0.52) or 2x (MANOVA, Wilks’ lambda: 0.93, F: 0.47, p = 0.83) experiments. There were significant differences in body size between SWNT treatment groups (MANOVA Wilks’ lambda: 0.21, F: 5.19, p = 0.001) and pairwise comparisons showed that spiders in the SWNT-CT group were statistically bigger than the control (p < 0.01) and the SWNT-T (p < 0.01) groups (Table 1).

**Table 1:** Morphological data for spiders in each treatment group for the three experiments.

Treatment Group	carapace width (cm)	body length (cm)	mass (g)
(1) GS			
Control (N = 12)	0.79 ± 0.15	3.52 ± 0.64	2.02 ± 1.27
MPT (N = 12)	0.86 ± 0.13	3.63 ± 0.45	1.96 ± 1.02
RPS (N = 12)	0.79 ± 0.17	3.36 ± 0.63	1.71 ± 0.79
(2) SWNT			
Control (N = 6)	1.11 ± 0.10	4.77 ± 0.31	4.78 ± 2.23
CT (N = 6)	1.12 ± 0.08	5.08 ± 0.28	5.21 ± 1.26
T (N = 6)	1.05 ± 0.19	4.53 ± 0.57	4.52 ± 2.11
(3) 2x			
Control (N = 14)	0.92 ± 0.16	3.85 ± 0.45	2.08 ± 0.77
CT (N = 14)	0.97 ± 0.10	3.96 ± 0.34	2.14 ± 0.63
MPT (N = 14)	0.96 ± 0.08	3.94 ± 0.34	2.18 ± 0.61

Average (mean ± 1 SD) values for body measurements of *N*. *pilipes* assigned to three treatment groups for each of the three experiments: (1) GS-Graphene Sheets, (2) SWNT-Single Walled Nanotubes and (3) 2x-Double Concentration.

### Silk mechanical properties

**Fig 1:**
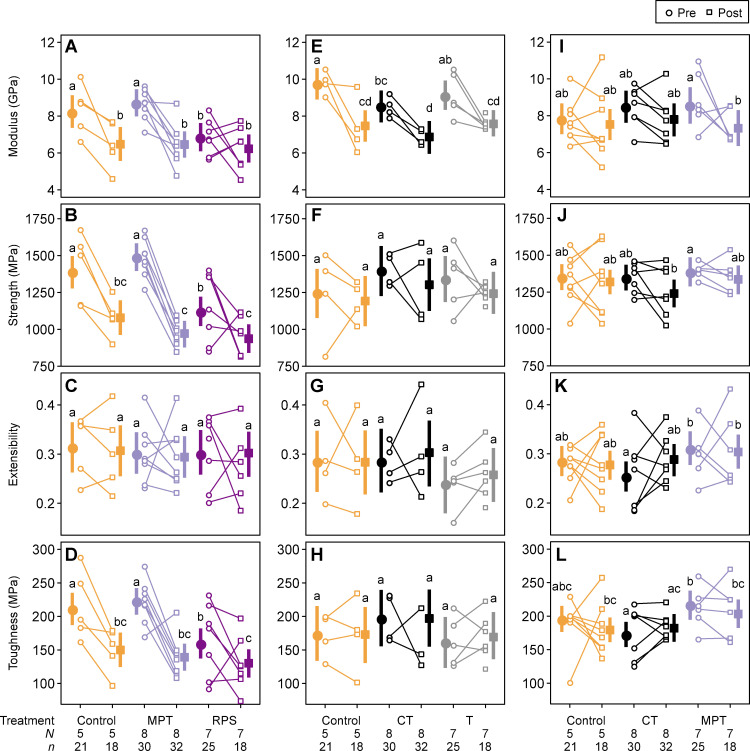
Mechanical properties of GS-Graphene Sheets (A-D), SWNT-Single-Walled Nanotubes (E-H) and 2x- Double Concentrations (I-L). Two empty symbols inked together indicates the empirical averages by individual spiders. Solid symbols and thick lines indicate the posterior means and their 95% highest density intervals. In each panel, significant differences between each of the 95% density intervals is represented by groups that do not share letters. Significant differences determined from multiple comparisons (S1 to S3 Tables).

From the original number of spiders in each group from the three experiments, there were some instances in which spiders died during the 10 days of the experiment and therefore the final number of individuals used for mechanical analyses were slightly different for each group from the three experiments. In each of the three experiments (GS, SWNT and 2x), there were different numbers of individual spiders (N) and numbers of pre-treatment and post-treatment silk samples (n) in the three treatment groups used. In the (1) GS experiment there were very little differences in extensibility but there were consistent decreases in modulus, strength and toughness between pre- and post-treatment silk samples for control and the two graphene groups (MPT and RS) (Fig 1A to 1C). Significant decreases were seen in all three groups in strength and toughness, and between control and MPT groups in modulus (Fig 1A to 1C). However, if focus is directed to just post-treatment silk, there were no significant differences between the control and the two experimental groups for any of the tensile properties (Fig 1A to 1C). In the (2) SWNT experiment the only significant differences between pre- and post-treatment silk samples was for modulus, in which there was a significant decrease in the control and the two SWNT groups (CT and T) (Fig 1D to 1F). However once again, if focus is directed to just post-treatment silk there were no significant differences between control and SWNT groups (Fig 1D to 1F). Results from the (3) 2x experiment were similar to that of the (2) SWNT experiment in which the only significant difference in tensile properties was a significant decrease in modulus, but only in the MPT group (Fig 1G to 1I). No significant differences were found between post-treatment silk from control and the two experimental groups (CT and MPT) for any of the tensile properties (Fig 1G to 1I).

**Fig 2:**
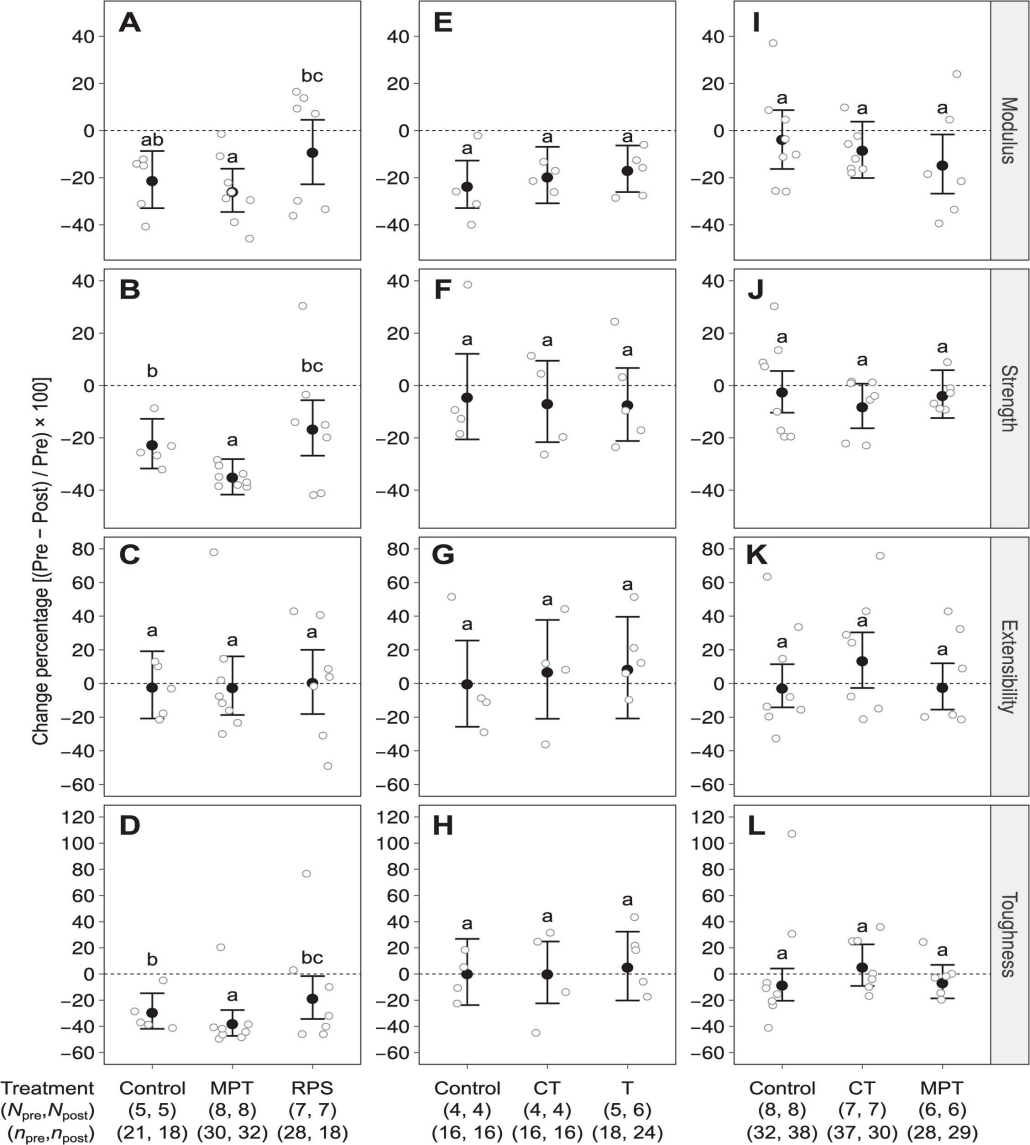
Percent change in mechanical properties (Young’s Modulus, Ultimate Strength, Extensibility and Toughness) between pre-treatment and post-treatment silk samples from the three rounds of experiments: GS-Graphene Sheets (A-D), SWNT-Single-Walled Nanotubes (E-H) and 2x- Double Concentrations (I-L). An empty circle indicates the average change percentage of an individual. Solid Circles and whiskers indicates the posterior means and their 95% highest density intervals. In each panel, significant differences between each of the 95% density intervals is represented by groups that do not share letters. Significant differences were determined from multiple comparisons (S4 to S6 Tables).

**Fig 3:**
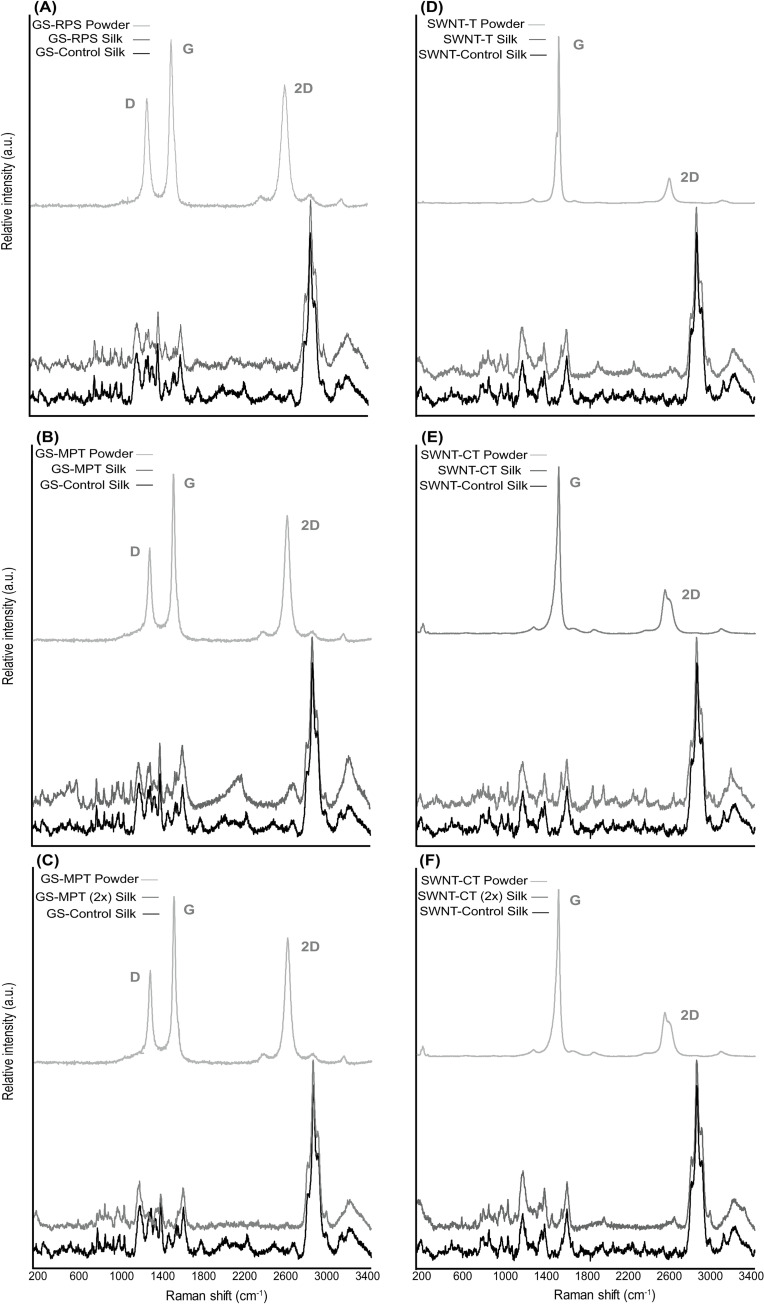
Raman spectroscopy analysis of silk samples and GS (MPT and RPS) and SWNT (CT and T) powders from the three rounds of experiments. 1) GS-Graphene Sheets (A, B), SWNT-Single-Walled Nanotubes (D, E) and 2x-Double Concentration (C, F).

Percent change in the tensile properties between pre-treatment and post-treatment silk samples of each treatment group in the three experiments did not indicate any changes between control and experimental groups (Fig 3). The only significant differences for percent changes in tensile properties were seen in the GS experiment between the two GS groups (MPT and RPS) for modulus, strength and toughness (Fig 3A, 3B and 3D). However, there were no significant differences between the control and either one of the GS groups (Fig 2).

### Raman spectroscopy

Raman spectra results indicated clear peaks in the GS and SWNT powder samples that are not evident in the treatment group silk samples. GS powder samples showed peaks for D bands at ~1400 cm^-1^, G bands at ~1600 cm^-1^ and 2D bands at ~2700 cm^-1^ (Fig 3A to 3C). In SWNT powder samples there were peaks for G bands at ~1600 cm^-1^ and 2D bands at ~2700 cm^-1^ (Fig 3D to 3F). There were no D, G or 2D bands of carbon nanomaterials evident in any of the silk samples. The peaks present in silk samples are associated with *n*(C-C) bonds of polypeptide chains located at ~1050–1150 cm^-1^, Amide groups (I, II, III) located from ~1200–1700 cm^-1^, along with amino acids (e.g., alanine, glycine, glutamine, proline) from ~2800–3100 cm^-1^ (Fig 3A to 3F). Similar peaks were seen in both control and experimental silk samples with no evident effects from GS or SWNT.

## Discussion

In our study, there were no significant improvements in tensile properties of MA silk collected from *N*. *pilipes* spiders that were fed either GS or SWNT. There were some significant decreases in tensile property values but because there were no significant differences between control and treatment groups when only post-treatment silk was considered we believe that any changes in tensile properties were not associated with the presence of carbon nanomaterials. In addition to no differences in post-treatment silk between control and experimental groups, we were also unable to detect the presence of GS or SWNT in silk samples using Raman spectroscopy analyses. Therefore, contrary to our predictions, there was no incorporation of GS or SWNTs into the MA silk of *N*. *pilipes* spiders. Our findings therefore contradict the proof of concept results that were discussed in the earlier study by Lepore et al. [19].

To the best of our knowledge there has only been one previously published study conducted that incorporated carbon nanomaterials in spider silk through the process of the spider ingesting solutions containing GS and CNTs [19]. There are a number of important aspects that differentiate our study with that of Lepore et al. [19]. 1) Effects on tensile properties that could be a result of using different spider species and different sexes were controlled for by usingonly adult females of *N*. *pilipes*. Additionally, morphological data for each individual was collected to control for the effects of body size on silk properties. 2) A control group was included in our study in addition to pre- and post-treatment silk samples in order to determine if there are any effects from possible artifacts impacting how the results are interpreted. 3) Single strands of MA silk were individually assessed for silk diameters allowing for accurate calculations of subsequent tensile properties. 4) Spiders were individually fed GS and SWNT solutions by holding the spider and directly pipetting the solution onto its chelicera. 5) Statistical analyses were used to test for significant differences between pre- and post-treatment samples, as well as between control and experimental groups. The modifications to our experimental design allowed for the reduction in the amount of variation that was seen in the Lepore et al. [19] study and to accurately determine whether carbon nanomaterials could be incorporated into the MA silk of the spider after being fed GS and SWNT solutions.

The Lepore et al. [19] study reported changes in silk strength and toughness ranging from mean values of increment changes ranging from -200 to 400%, along with max values ranging from 300–600%. In our study we found far less variation in percent change in pre- and post-treatment silk samples with minimum and maximum values ranging from ~ -50 to 120 respectively (Fig 3). One important aspect of our study in contrast to the Lepore et al. [19] study was the inclusion of a control group for each of the three experiments. This allowed us to determine that although there were some significant changes between pre- and post-treatment samples for some of the tensile properties, there was no overall difference between control and experimental groups. In addition to the increases in mechanical properties, Lepore et al. [19] also showed evidence for the presence of GS and SWNT in silk samples using Raman spectroscopy analyses. However, from the description of the methods, it seemed possible that the positive results from Raman analyses were from contamination of silk fibers during the initial spraying of carbon nanomaterial solutions. We found no evidence for the presence of GS or SWNT in our study in which we fed spiders by pipette and therefore removed the possibility for sample contamination with carbon nanomaterial solutions.

Other previous studies involving the combination of spider silk and carbon nanomaterials were focused on coating the exterior of the silk, instead of attempting to have the spiders ingest GS or CNT solutions [3, 20]. In a study by Steven et al., (2013) the silk of *Nephila clavipes* was coated with amine functionalized multiwall CNTs (f-CNTs) and although there was no improvement in overall toughness, there was clear evidence for improving the conductivity of the fiber. Li et al., [3] coated the silk of *Nephila clavita* using a graphene oxide-based layer-by-layer method, in which they were able to create a highly sensitive fiber that produced electrical signals when deformed by even slight environmental disturbances (e.g., movement of an ant, throat vibrations when someone speaks). They were successful in fabricating a tendril-like strain sensor, as well as a humidity and vibration sensor which could have great potential for wearable electronics [3]. However, Li et al., [3] did not test for tensile properties of the silk coated fiber. In summary, the use of spider silk coated with carbon nanomaterials seems to not improve the tensile properties of the silk, but there is strong evidence for it being an exciting prospect for creating highly conductive fibers that retain their initial toughness.

## Conclusion

In conclusion, the results from this study showed that the attempts to improve upon the tensile properties of *N*. *pilipes* MA silk by feeding them solutions with carbon nanomaterials was not successful. Although similar methods were used as to those of Lepore et al., [19], the results discussed in their work could not be replicated. The incorporation of carbon nanomaterials into the protein matrix of spider silk will allow for novel research in the field of biomechanics that can investigate the mechanical properties of a biosynthetic material new to science. However, as was observed in this study, there is still some doubt on the possibility for carbon nanomaterials to be incorporated into the silk matrix simply by feeding the spider solutions containing GS and CNTs. Additional research will be necessary in attempting to validate the findings reported within the Lepore et al., [19] study before it will be possible to make any significant advances in the creation of new biomaterials involving carbon nanomaterials incorporated within the spider silk matrix. We believe that our study represents an example for the importance of attempting to replicate previously published research. Researchers should be encouraged to continue to do these types of investigations to build a strong consensus and solid foundation for how to go forward with these exciting new methods for creating novel biomaterials.

## Supporting information

S1 Table Multiple comparisons of GS-Graphene Sheets data set. (DOCX)

S2 Table Multiple comparisons of SWNT-Single-Walled Nanotubes data set. (DOCX)

S3 Table Multiple comparisons of 2x- double concentrations data set. (DOCX)

S4 Table Multiple comparisons of change percentages of GS-Graphene Sheets data set data set. (DOCX)

S5 Table Multiple comparisons of change percentages of SWNT-Single-Walled Nanotubes data set. (DOCX)

S6 Table Multiple comparisons of change percentages of 2x- double concentrations data set. (DOCX)
