# Supplementary material for: Mechanical and structural properties of major ampullate silk from spiders fed carbon nanomaterials
Source: PLoS One. 2020 Nov 9;15(11):e0241829. doi: 10.1371/journal.pone.0241829 (PMC7652353; doi:10.1371/journal.pone.0241829)
Supplement: S1 Table — (DOCX) [file pone.0241829.s001.docx]

**S1 Table. Multiple comparisons of GS-Graphene Sheets data set.** Posterior differences of modulus (A), strength (B), extensibility (C) and toughness (D) are showed in lower triangle, and posterior effect size (i.e. posterior difference over residual standard deviation) are listed in upper triangle. Range inside parentheses denotes the 95% highest density interval. Numbers in bold indicate a significant difference, which means the corresponding 95% HDI of posterior effect size is completely laying outside of ±0.1.

| Tensile properties | Subtrahend | Minuend | | | | | |
| --- | --- | --- | --- | --- | --- | --- | --- |
|  |  | Control-Pre | CT-Pre | MPT-Pre | Control-Post | CT-Post | MPT-Post |
| (A) |  |  |  |  |  |  |  |
| Modulus (GPa) | Control-Pre | ——— | −5.00 (−16.39, 6.68) | **13.67 (2.11, 25.75)** | **17.55 (6.33, 29.02)** | **17.67 (5.51, 29.13)** | **20.06 (8.21, 32.78)** |
|  | CT-Pre | −0.50 (−1.60, 0.66) | ——— | **18.67 (8.05, 30.07)** | **22.55 (10.45, 35.13)** | **22.67 (13.16, 32.48)** | **25.06 (14.13, 36.26)** |
|  | MPT-Pre | **1.35 (0.22, 2.51)** | **1.85 (0.77, 2.87)** | ——— | 3.87 (−8.23, 16.04) | 3.99 (−6.92, 14.41) | 6.38 (−3.37, 16.48) |
|  | Control-Post | **1.73 (0.62, 2.80)** | **2.23 (1.02, 3.38)** | 0.38 (−0.79, 1.58) | ——— | 0.12 (−11.38, 11.94) | 2.51 (−9.63, 14.33) |
|  | CT-Post | **1.75 (0.65, 2.91)** | **2.24 (1.35, 3.15)** | 0.39 (−0.63, 1.45) | 0.01 (−1.16, 1.13) | ——— | 2.39 (−8.25, 13.02) |
|  | MPT-Post | **1.98 (0.81, 3.14)** | **2.48 (1.47, 3.55)** | 0.63 (−0.34, 1.61) | 0.25 (−0.95, 1.40) | 0.24 (−0.78, 1.30) | ——— |
| (B) |  |  |  |  |  |  |  |
| Strength (MPa) | Control-Pre | ——— | −5.22 (−12.79, 2.16) | **14.20 (6.37, 22.28)** | **16.52 (8.52, 24.53)** | **22.17 (13.84, 30.12)** | **24.01 (15.66, 32.85)** |
|  | CT-Pre | −99.19 (−241.10, 40.53) | ——— | **19.42 (11.91, 27.12)** | **21.74 (13.49, 30.28)** | **27.39 (20.14, 34.57)** | **29.23 (21.25, 37.44)** |
|  | MPT-Pre | **269.14 (123.25, 415.67)** | **368.33 (235.41, 507.59)** | ——— | 2.32 (−6.08, 10.23) | **7.97 (0.89, 15.16)** | **9.81 (2.79, 16.74)** |
|  | Control-Post | **313.12 (164.86, 455.10)** | **412.31 (265.28, 564.32)** | 43.99 (−113.61, 193.63) | ——— | 5.65 (−1.91, 13.49) | 7.49 (−0.71, 15.24) |
|  | CT-Post | **420.26 (277.61, 564.66)** | **519.45 (401.64, 638.77)** | **151.12 (16.71, 282.77)** | 107.13 (−40.37, 250.10) | ——— | 1.84 (−5.00, 9.17) |
|  | MPT-Post | **455.05 (302.72, 598.46)** | **554.24 (422.96, 691.59)** | **185.91 (60.41, 318.85)** | 141.92 (−3.15, 295.47) | 34.79 (−92.01, 174.36) | ——— |
| (C) |  |  |  |  |  |  |  |
| Extensibility | Control-Pre | ——— | 2.46 (−10.03, 14.92) | 2.55 (−9.02, 16.30) | 1.38 (−10.62, 13.57) | 3.88 (−8.61, 16.69) | 2.31 (−11.04, 15.39) |
|  | CT-Pre | 0.01 (−0.05, 0.08) | ——— | 0.09 (−11.87, 11.71) | −1.09 (−14.00, 11.80) | 1.41 (−8.98, 11.26) | −0.15 (−12.08, 11.80) |
|  | MPT-Pre | 0.01 (−0.05, 0.08) | 0.00 (−0.06, 0.06) | ——— | −1.17 (−13.79, 12.33) | 1.33 (−10.63, 13.25) | −0.24 (−10.63, 11.21) |
|  | Control-Post | 0.01 (−0.05, 0.07) | −0.01 (−0.07, 0.06) | −0.01 (−0.07, 0.06) | ——— | 2.50 (−10.42, 15.36) | 0.93 (−12.48, 13.79) |
|  | CT-Post | 0.02 (−0.04, 0.09) | 0.01 (−0.04, 0.06) | 0.01 (−0.06, 0.07) | 0.01 (−0.05, 0.08) | ——— | −1.57 (−13.48, 10.30) |
|  | MPT-Post | 0.01 (−0.06, 0.08) | −0.00 (−0.06, 0.06) | −0.00 (−0.06, 0.05) | 0.00 (−0.06, 0.07) | −0.01 (−0.07, 0.05) | ——— |
| (D) |  |  |  |  |  |  |  |
| Toughness (GPa) | Control-Pre | ——— | −2.49 (−9.17, 4.22) | **10.97 (4.05, 18.15)** | **13.09 (5.78, 20.14)** | **15.37 (8.49, 22.90)** | **17.28 (10.18, 24.58)** |
|  | CT-Pre | −11.69 (−43.51, 18.69) | ——— | **13.45 (6.73, 20.32)** | **15.58 (7.84, 22.69)** | **17.85 (11.87, 24.23)** | **19.77 (12.83, 26.55)** |
|  | MPT-Pre | **51.49 (20.05, 84.50)** | **63.18 (33.30, 93.44)** | ——— | 2.12 (−5.15, 9.59) | 4.40 (−2.24, 10.74) | **6.32 (0.15, 12.53)** |
|  | Control-Post | **61.37 (27.48, 91.93)** | **73.06 (39.74, 105.57)** | 9.88 (−23.60, 44.89) | ——— | 2.28 (−4.83, 9.02) | 4.19 (−2.60, 11.44) |
|  | CT-Post | **72.10 (41.15, 104.21)** | **83.79 (58.04, 110.40)** | 20.61 (−8.53, 51.35) | 10.73 (−22.29, 42.43) | ——— | 1.92 (−4.52, 7.94) |
|  | MPT-Post | **81.10 (48.79, 112.58)** | **92.79 (64.24, 122.24)** | **29.61 (1.05, 57.89)** | 19.73 (−13.86, 51.99) | 9.00 (−20.01, 38.06) | ——— |
