# Supplementary material for: Mechanical and structural properties of major ampullate silk from spiders fed carbon nanomaterials
Source: PLoS One. 2020 Nov 9;15(11):e0241829. doi: 10.1371/journal.pone.0241829 (PMC7652353; doi:10.1371/journal.pone.0241829)
Supplement: S6 Table — (DOCX) [file pone.0241829.s006.docx]

**S6 Table. Multiple comparisons of change percentages of 2x- Double Concentrations data set.** Posterior differences of pre-post effect of (A) modulus, (B) strength, (C) extensibility and (D) toughness are showed in lower triangle, and posterior effect size (i.e. posterior difference over residual standard deviation) are listed in upper triangle. Range inside parentheses denotes the 95% highest density interval.

| Tensile properties | Subtrahend | Minuend | | |
| --- | --- | --- | --- | --- |
|  |  | Control | CT | MPT |
| (A) |  |  |  |  |
| Modulus | Control | ——— | −3.42 (−14.95, 8.51) | −7.92 (−21.09, 3.94) |
|  | CT | 4.64 (−12.87, 21.36) | ——— | −4.5 (−17.5, 8.28) |
|  | MPT | 10.92 (−6.13, 28.89) | 6.29 (−12.04, 22.64) | ——— |
| (B) |  |  |  |  |
| Strength | Control | ——— | −3.5 (−10.81, 3.65) | −0.92 (−8.61, 6.57) |
|  | CT | 5.66 (−5.7, 17.09) | ——— | 2.58 (−5.56, 10.34) |
|  | MPT | 1.36 (−9.98, 13.79) | −4.3 (−16.59, 8.23) | ——— |
| (C) |  |  |  |  |
| Extensibility | Control | ——— | 6.74 (−1.85, 15.72) | 0.13 (−8.76, 8.9) |
|  | CT | −16.27 (−37.23, 4.84) | ——— | −6.61 (−16.16, 2.84) |
|  | MPT | −0.48 (−18.03, 18.56) | 15.8 (−6.38, 37.3) | ——— |
| (D) |  |  |  |  |
| Toughness | Control | ——— | 4.62 (−1.83, 11.33) | 0.39 (−6.45, 7.14) |
|  | CT | −13.76 (−34.91, 5.22) | ——— | −4.24 (−10.97, 3.02) |
|  | MPT | −1.81 (−18.67, 16.52) | 11.95 (−8.5, 32.22) | ——— |
